# A case of concomitant clinical and histopathologic features of lymphocytic thrombophilic arteritis and livedoid vasculopathy

**DOI:** 10.1016/j.jdcr.2023.08.033

**Published:** 2023-09-09

**Authors:** Christian Gan, Daryl Johnson, Christopher McAulay-Powell, Robert Kelly

**Affiliations:** a Department of Dermatology, St Vincent’s Hospital, Melbourne, Australia; b Department of Pathology, St Vincent’s Hospital, Melbourne, Australia; c Department of Neurology, Barwon Health, Geelong, Australia

**Keywords:** livedo racemosa, livedoid vasculopathy, lymphocytic thrombophilic arteritis, macular lymphocytic arteritis, vasculopathy

## Introduction

Lymphocytic thrombophilic arteritis and livedoid vasculopathy are lymphocytic conditions that may both present with livedo racemosa and ulceration.^1^, ^2^, ^3^, ^4^, ^5^ We present a case with clinical and histologic features of both conditions, raising the possibility that these conditions are either closely linked or are part of a spectrum of the same condition. Lymphocytic thrombophilic arteritis and livedoid vasculopathy have characteristic clinical and histologic features.^1^, ^2^, ^3^, ^4^, ^5^ Livedoid vasculopathy presents with small, superficial ulcers that most commonly occur over the ankles, shins, and feet, whereas livedo racemosa may occur in up to 79% of cases.^3^, ^4^, ^5^ Lymphocytic thrombophilic arteritis typically presents with widespread livedo racemosa over the upper and lower limbs, buttocks, and lower trunk.^1^^,^^2^ A distinct pattern of ulceration similar to that seen in livedoid vasculopathy and macular pigmentation may also occur.^1^^,^^6^^,^^7^ In livedoid vasculopathy, the histology shows extensive fibrin deposition in the walls and lumen of papillary dermal vessels and a variable light perivascular lymphocytic infiltrate.^3^^,^^4^ In lymphocytic thrombophilic arteritis, there is a dense perivascular lymphocytic infiltrate around small- to medium-sized arteries in the reticular dermis and upper subcutis with prominent luminal and mural fibrin deposition.^1^^,^^2^ A luminal fibrin ring may be seen in some but not all cases and is not essential for the diagnosis.^1^ Neuropathy may be seen in both conditions.^1^^,^^8^

## Case report

**Fig 1:**
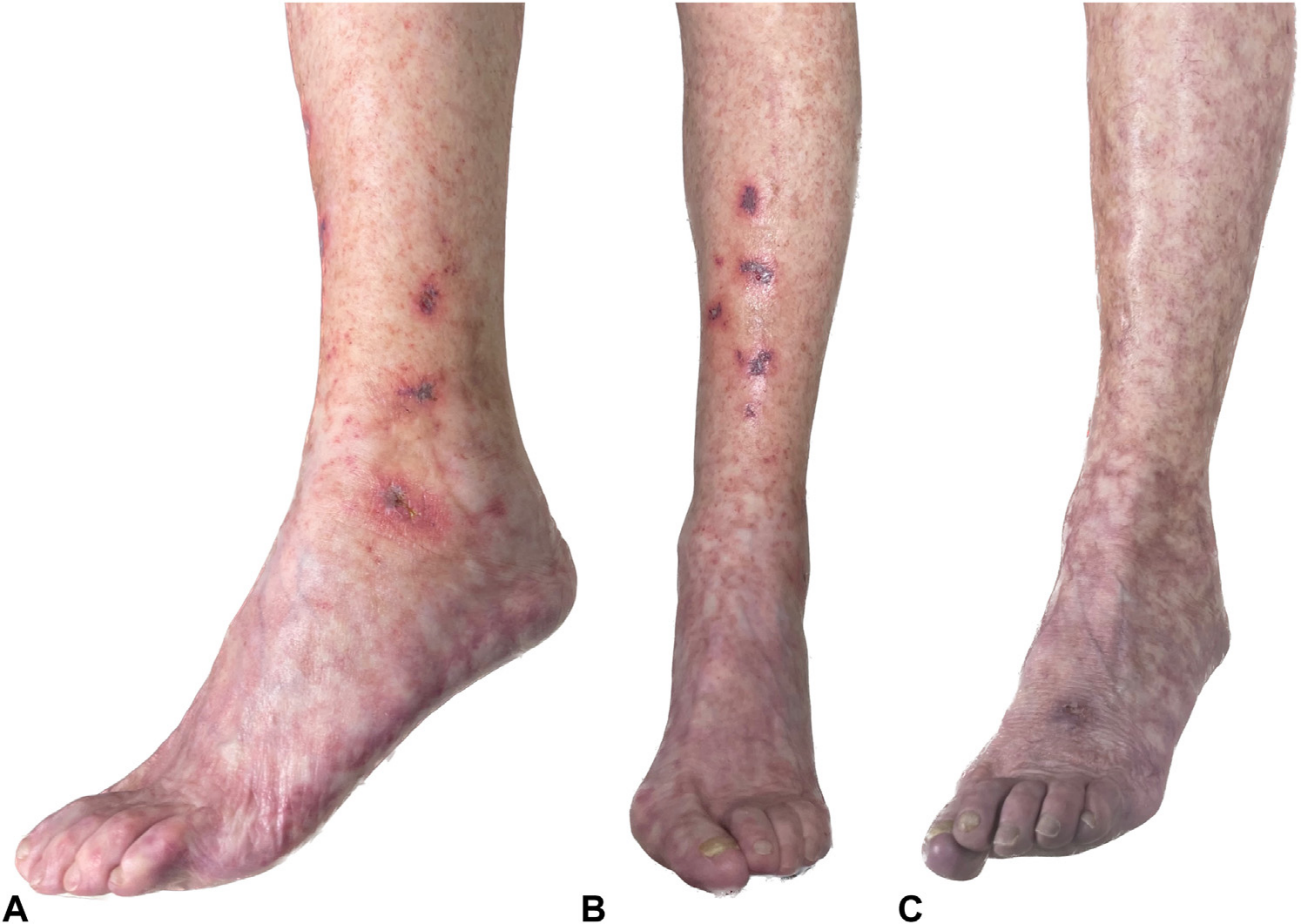
**A, B,** Superficial small, crusted ulcers over the left ankle area and shin on a background of extensive livedo racemosa. **C,** Resolution of ulcerations with residual livedo racemosa 4 weeks following treatment with rivaroxaban.

A 68-year-old woman presented with a 5-year history of widespread livedo racemosa over her upper and lower limbs, buttocks, and lower trunk. She had a 2-year history of recurrent painful bilateral ulcerations that were prominent over her ankles and shins that would self-resolve over approximately 2 months and would recur every 2 to 3 months. They had a noninflammatory appearance and were covered with a superficial dark crust (Fig 1). She also had a 10-year history of a symmetric glove and stocking peripheral neuropathy, which was not consistent with mononeuritis multiplex and thought to be unrelated to her current condition.

**Fig 2:**
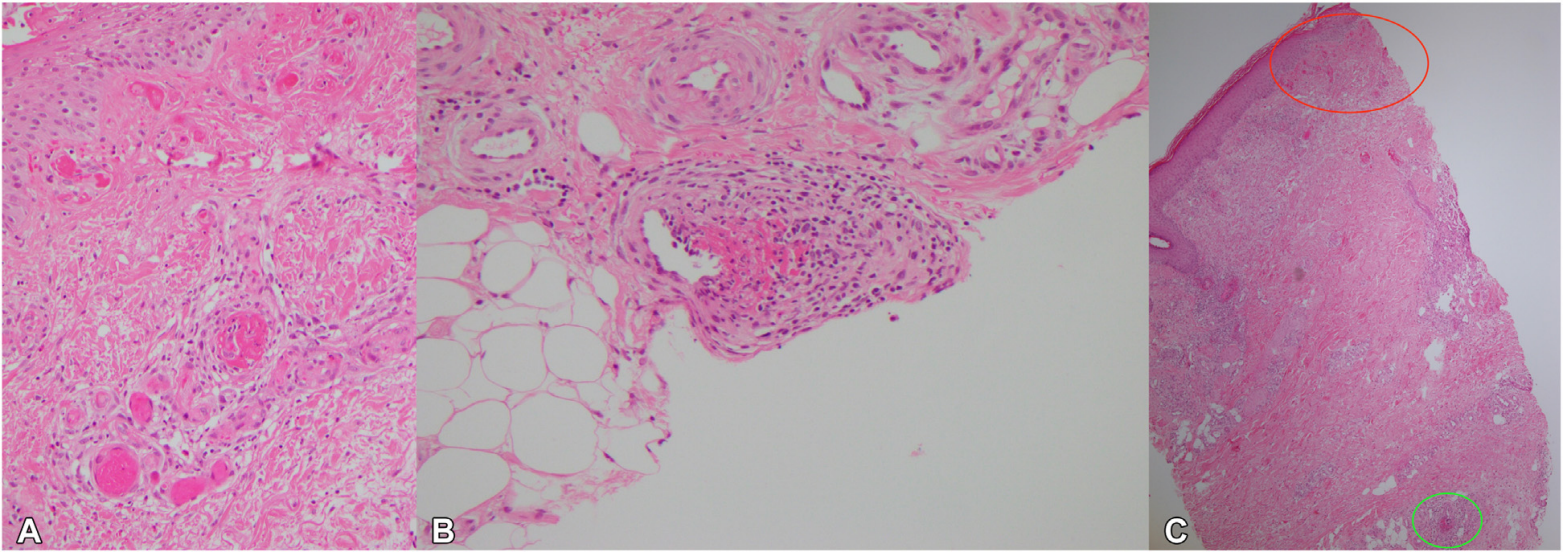
**A,** Extensive fibrin deposition in the wall and lumen of small papillary dermal vessels with a light perivascular lymphocytic inflammation consistent with livedoid vasculopathy. **B,** Dense lymphocytic inflammation around vessel in the reticular dermis with fibrin deposition consistent with lymphocytic thrombophilic arteritis. **C,** Low-power photomicrograph showing features of both livedoid vasculopathy in superficial vessels (*red*) and features of lymphocytic thrombophilic arteritis in deeper vessel (*green*). (Original magnification: ×40.)

The histology adjacent to the ulcers showed diagnostic features of livedoid vasculopathy in the papillary dermis. There was extensive fibrin deposition in the walls and lumen of papillary dermal vessels and a light perivascular lymphocytic infiltrate (Fig 2). Changes consistent with lymphocytic thrombophilic arteritis were present in the reticular dermis and upper subcutis, where there was a dense perivascular lymphocytic infiltrate around small- to medium-sized arteries with prominent luminal and mural fibrin deposition (Fig 2).

Investigations showed evidence of a prothrombin 20210A heterozygous gene mutation. Her inflammatory, autoimmune, and other thrombophilic markers were unremarkable.

Her ulcers improved with aspirin 100 mg/d and pentoxifylline 400 mg 3 times a day. She developed pruritus secondary to pentoxifylline, which was ceased, after which the ulcers recurred. Rivaroxaban 20 mg/d was then commenced with complete resolution of all ulcers at 4 weeks, which remained clear at a 4 months review.

## Discussion

Lymphocytic thrombophilic arteritis, also known as macular lymphocytic arteritis, was initially described in 2003.^7^ It is a different condition from cutaneous polyarteritis nodosa, which is neutrophilic and has different clinical and histologic features (Table I). The differences have been highlighted in a study involving detailed clinicopathologic correlation.^1^ Confusion between lymphocytic thrombophilic arteritis and cutaneous polyarteritis nodosa has potentially impeded an understanding of lymphocytic thrombophilic arteritis as well as its relationship with livedoid vasculopathy.^9^ The simultaneous occurrence of these 2 conditions has also been noted previously in a study of 6 cases.^10^

**Table I:** The differences in clinical features, histopathologic findings, and treatment response between lymphocytic thrombophilic arteritis and cutaneous polyarteritis nodosa^10^

Characteristic	Lymphocytic thrombophilic arteritis	Cutaneous polyarteritis nodosa
Clinical features		
	Widespread Livedo racemosa Macular pigmentation	Localized patches of starburst livedo racemosa Tender nodules
	Noninflammatory punctate superficial ulceration	Larger inflammatory ulceration
	Chronic indolent course	Relapsing-remitting disease course
	Peripheral neuropathy	Peripheral neuropathy Arthralgias, myalgias.
Histologic differences	Dense lymphocytic inflammation prominent vascular fibrin deposition in small- to medium-sized arteries in reticular dermis and upper subcutis. Smaller more superficial vessels may also be involved.	Neutrophilic inflammation with fibrinoid necrosis in small to medium-sized arteries in the reticular dermis and upper subcutis. Lymphohistiocytic infiltrate in subacute lesions.
Treatment	Treatment targeting vascular fibrin deposition including asprin, pentoxifylline, and novel oral anticoagulants.	Treatment with immunosuppressive or anti-inflammatory agents, including prednisolone, colchicine, and steroid-sparing agents.

Livedoid vasculopathy has generally been thought to be a thrombo-occlusive vasculopathy affecting predominantly small vessels in the papillary dermis, giving rise to the characteristic small superficial ulcers.^3^, ^4^, ^5^ A light lymphocytic infiltrate, however, may also be present,^3^, ^4^, ^5^ raising the possibility that immune factors may also play a role in the pathogenesis. It nevertheless generally responds to therapies targeting vascular fibrin deposition whereas anti-inflammatory or immunosuppressive therapies have generally been ineffective, supporting a predominantly thrombophilic pathogenesis.^3^, ^4^, ^5^ Lymphocytic thrombophilic arteritis is considered to be a lymphocytic arteritis with prominent fibrin deposition giving rise to livedo racemosa, although smaller vessel involvement has been noted before.^1^^,^^6^ It can also respond to treatments targeting vascular fibrin deposition. Increased incidence of serologic markers of thrombophilia has also been noted in both conditions.^1^^,^^10^ The positive prothrombin 20210A heterozygous gene mutation was thus likely to be a pathogenic factor in this case. The presence of a dense vascular lymphocytic inflammation would also suggest that immunologic as well as thrombophilic factors are pathogenic in lymphocytic thrombophilic arteritis, although thrombophilia is a more important target therapeutically.^1^^,^^8^

The question arises as to why deeper vessel involvement has not been described previously in livedoid vasculopathy, particularly given the presence of livedo racemosa, which is a manifestation of larger deeper vessel involvement. This may relate to the use of superficial biopsies capturing superficial changes but missing deeper pathology and sampling-related issues, given that deeper biopsies may miss unevenly distributed vascular pathology. To capture this pathology, multiple biopsies and serial sectioning may be required.

This case, along with previous reports, raises the possibility that lymphocytic thrombophilic arteritis and livedoid vasculopathy are at least very closely related or even a spectrum of the same condition with livedo racemosa being the dominant finding in lymphocytic thrombophilic arteritis and ulceration being the dominant finding in livedoid vasculopathy.

## Conflicts of interest

None disclosed.
